# 

**Published:** 1893-02

**Authors:** 


### BOOKS RECEIVED.

The Anatomy and Surgical Treatment of Hernia. By Henry O. Marcy, A. M., M. D., LL. D., of Boston. President of the American Medical Association; Surgeon of the Hospital for Women, Cambridge;

President of the Section of Gynecology, Ninth International Congress; Late President of the American Academy of Medicine; Member of the British Medical Association; Member of the Massachusetts Medical Society; Honorary Member of the New York State Medical Association; Fellow of the Boston Gynecological Society; Corresponding Member of the Medico-Chirurgical Society of Bologna, Italy; Fellow of the American Association of Obstetricians and Gynecologists; Fellow of the Southern Surgical and Gynecological Association; Late Surgeon U. S. Army, etc. With sixty-six full-page heliotype and lithographic plates, including eight colored plates from Bougery, and thirty-seven illustrations in the text. New York: D. Appleton & Co. 1892. Sold only by subscription. Half morocco. $15.00.

Notes of the Newer Remedies, their Therapeutic Applications and Modes of Administration. By David Cerna, M. D., Ph. D., Demonstrator of Physiology in the Medical Department of the University of Texas, Galveston; formerly Assistant in Physiology, Demonstrator of, and Lecturer on, Experimental Therapeutics in the University of Pennsylvania; Fellow of the College of Physicians of Philadelphia; Corresponding Fellow of the Sociedad Española de Hygiene, of Madrid; Associate Editor of Sajous' Annual of the Universal Medical Sciences, etc. Duodecimo, pp. viii —177. Price, $1 25. Philadelphia: W. B. Saunders, 913 Walnut street. 1893.

A Text-Book of Practical Therapeutics, with especial reference to the application of remedial measures to disease, and their employment upon a rational basis. By Hobart Amory Hare, M. D., B. Sc., Professor of Therapeutics and Materia Medica in the Jefferson Medical College of Philadelphia; Laureate of the Royal Academy of Medicine in Belgium, of the Medical Society of London, etc.; President of the Section of Therapeutics in the Pan-American Medical Congress. Third edition. Enlarged and thoroughly revised. Cloth, $3.75; leather, $4.75. Philadelphia: Lea Brothers & Co. 1892.

A Manual of Ophthalmology. By Howard F. Hansell, M. D., Lecturer on Ophthalmology in the Jefferson Medical College Hospital, Chief Clinical Assistant in Eye Department, Jefferson Medical College Hospital; Member of the American Ophthalmological Society; Fellow of the College of Physicians, Philadelphia, etc.; and James H. Bell, M. D., lately Demonstrator of Anatomy in Jefferson Medical College; Member of Ophthalmological staff, Jefferson Medical College Hospital; Ophthalmic Surgeon to the Southwestern Hospital, and Dispensary, etc. With 120 illustrations. Price, $1.75. Duodecimo, pp. xiv.—231. Philadelphia; P. Blakiston, Son & Co., 1012 Walnut street. 1892.

Transactions of the Medical Society of the State of North Carolina. Thirty-ninth annual session, held at Wilmington, N. C., May 17, 18, and 19, 1892. Wilmington, N. C.: Jackson & Bell, steam power presses. 1892.

---

Notice to Contributors.—We are glad to receive contributions from every one who knows anything of interest to the profession. Articles designed for publication in the Journal should be handed in before the first day of the month. The Editors are not responsible for the views or opinions of contributors. All communications should be addressed to the Managing Editor, 284 Franklin St., Buffalo, N. Y.

